# FAK is Required for Tumor Metastasis-Related Fluid Microenvironment in Triple-Negative Breast Cancer

**DOI:** 10.3390/jcm8010038

**Published:** 2019-01-02

**Authors:** Mei-Ren Pan, Ming-Feng Hou, Fu Ou-Yang, Chun-Chieh Wu, Shu-Jyuan Chang, Wen-Chun Hung, Hon-Kan Yip, Chi-Wen Luo

**Affiliations:** 1 Graduate Institute of Clinical Medicine, Kaohsiung Medical University, Kaohsiung 807, Taiwan; mrpan@cc.kmu.edu.tw (M.-R.P.); mifeho@kmu.edu.tw (M.-F.H.); 2 Department of Surgery, Kaohsiung Medical University Hospital, Kaohsiung 807, Taiwan; kmufrank@gmail.com; 3 Division of Breast Surgery, Department of Surgery, Kaohsiung Medical University Hospital, Kaohsiung 807, Taiwan; 4 Department of Pathology, Kaohsiung Medical University Hospital, Kaohsiung Medical University, Kaohsiung 807, Taiwan; lazzz_wu@yahoo.com.tw; 5 Graduate Institute of Medicine, College of Medicine, Kaohsiung Medical University, Kaohsiung 807, Taiwan; binbin4728@hotmail.com; 6 National Institute of Cancer Research, National Health Research Institutes, Tainan 704, Taiwan; hung1228@nhri.org.tw; 7 Division of Cardiology, Department of Internal Medicine, Kaohsiung Chang Gung Memorial Hospital, Kaohsiung 807, Taiwan; 8 Institute for Translational Research in Biomedicine, Kaohsiung Chang Gung Memorial Hospital, Kaohsiung 807, Taiwan; 9 Center for Shockwave Medicine and Tissue Engineering, Kaohsiung Chang Gung Memorial Hospital, Kaohsiung 807, Taiwan

**Keywords:** Fluid shear stress, focal adhesion kinase, triple-negative breast cancer, cell migration, drug sensitivity

## Abstract

Cancer cell metastasis is the main cause of death in patients with cancer. Many studies have investigated the biochemical factors that affect metastasis; however, the role of physical factors such as fluid shear stress (FSS) in tumorigenesis and metastasis have been less investigated. Triple-negative breast cancer (TNBC) has a higher incidence of lymph node invasion and distant metastasis than other subtypes of breast cancer. In this study, we investigated the influence of FSS in regulating the malignant behavior of TNBC cells. Our data demonstrate that low FSS promotes cell migration, invasion, and drug resistance, while high FSS has the opposite results; additionally, we found that these phenomena were regulated through focal adhesion kinase (FAK). Using immunohistochemistry staining, we show that FAK levels correlate with the nodal stage and that FAK is a significant independent predictor of overall survival in patients. Altogether, these data implicate FAK as a fluid mechano-sensor that regulates the cell motility induced by FSS and provide a strong rationale for cancer treatments that combine the use of anti-cancer drugs and strategies to modulate tumor interstitial fluid flow.

## 1. Introduction

Cancer is the leading cause of death worldwide. However, most cancer-related deaths are not caused by the primary tumors, but by metastases. Metastatic cells undergo biological and physical changes through several steps, such as detachment, intravasation, circulation in the bloodstream, extravasation, and colonization [1]. Prior studies have focused mostly on the biochemical factors that affect metastasis formation; however, the effect of several physical factors on this process has been less investigated. Increasing evidence has indicated that mechanical stress plays an important role not only in tissue development and maintenance, but also in the pathogenesis of several diseases, including cancer [2]. The fluid flow in and around the tumor tissues affects the extracellular gradient of chemokines and growth factors and plays a role in the delivery of anti-cancer drugs [3,4,5]. However, the influence of flow-induced stress on tumor cell biology and malignancy has been poorly investigated.

Fluid shear stress (FSS), one of the stresses induced by liquid flow, such as blood or interstitial flow, is pervasive in all living tissues. Prior studies have indicated that FSS affects cytokine production and immune cell adhesion in lymphatic and venous vasculatures [6]. In addition, in adherent cancer cells, FSS enhances G_2_/M arrest [7], regulates cell death by autophagy and apoptosis [8], promotes phenotypic changes associated with transformation and progression [9], and affects irradiation sensitivity [10]. Furthermore, several groups have recently found that FSS stimulates migration and extravasation of tumor cells by inducing oxidative stress [11] and alters the viability and proliferation of circulating cancer cells (CTCs) [12]. This evidence indicates that FSS is an important regulator in both adherent and circulating tumor cells.

Triple-negative breast cancer (TNBC), one of the subtypes of breast cancer, accounts for 15–20% of all breast cancer cases [13], and is characterized by loss of the expression of the estrogen receptor (ER), progesterone receptor (PR), and human epidermal growth factor receptor 2 (HER2) [14]. There are no approved targeted therapies for patients with TNBC; the therapy, in such cases, is merely systemic chemotherapy. A high metastatic rate is the prominent cause of the high mortality in patients with TNBC [15]. Previous studies have indicated that anti-angiogenic agents could normalize the blood vessels and improve the blood flow, therefore normalizing FSS [16,17,18,19]. This effect might sensitize the tumor to radiotherapy and increase its exposure to cytotoxic chemotherapy [10,16,17,18]. Many investigators have studied the biological effects of the combination of anti-angiogenic agents and chemo- or radio-therapy on TNBC [20,21,22]; however, the role of FSS in regulating drug sensitivity and cellular functions in cancer cells is not yet understood.

Focal adhesion kinase (FAK), a 125-kDa non-receptor protein tyrosine kinase, regulates several cellular functions such as survival, invasion, motility, adhesion, metastasis, proliferation, and angiogenesis in normal and cancer cells [23,24,25]. Overexpression of FAK has been observed in a variety of tumors [23,24,26,27,28,29]. A previous study indicated that high FAK levels are associated with lymphovascular invasion and a triple-negative phenotype in breast cancer [30]. Activation of FAK induces epithelial-mesenchymal transition (EMT), migration, and invasion in TNBC cells, while the inhibition of FAK targets cancer stem cells and blocks the metastatic ability in breast cancer cells [23,31]. Our previous study indicated that knockdown of FAK increases the cytotoxicity of irradiation in colorectal cells after FSS stimulation [10]. Similarly, other studies have indicated that FAK participates in FSS-induced cell motility, adhesion, and metastasis in several types of cancer [3,32,33,34]. However, whether FAK also affects FSS-induced cellular functions in TNBC cells is still unclear.

Therefore, the aims of this study were to investigate whether FSS regulates the malignancy of TNBC cells via the FAK-mediated signaling pathway and to clarify the role of FAK in patients with TNBC.

## 2. Materials and Methods

### 2.1. Parallel-Plate Flow Chamber and Circulatory System

A parallel-plate flow chamber (PPFC) was used to induce FSS in cultured cells, as described previously [7,8,10]. In brief, a silicone gasket was sandwiched between a glass slide and an acrylic plate to create a flow channel, therefore exposing the cells to FSS. The cells were seeded on the slides, which were pre-coated with fibronectin (Thermo Fisher Scientific, Waltham, MA, USA). A laminar flow was generated by a peristaltic pump. In this system, FSS (τ) = 6μQ/bh^2^, where Q is the flow rate (cm^3^/s), μ is the fluid viscosity (0.01 dyn-s/cm^2^), and b and h are the width (mm) and height (mm), respectively. For this study, we modified our PPFC system to a circulatory one [11]. A suspension of cells, obtained upon trypsinization of cells grown on a culture dish, was injected into the circulatory system. FSS was calculated using Poiseuille’s equation, τ = 4μQ/πR^3^, where τ is the FSS (dyn/cm^2^), Q is the flow rate (cm^3^/s), μ is the viscosity of the fluid, and R is the radius of the tube (mm). The chamber and circulating system were kept at 37 °C and equilibrated with 95% humidified air with 5% CO_2_.

### 2.2. Cell Culture

In this study, we used the TNBC cancer cell lines MDA-MB-231 and MDA-MB-468 (ATCC, Manassas, VA, USA). Cells were maintained in Dulbecco’s modified Eagle’s medium (DMEM) or DMEM/F12 medium (both from Thermo Fisher Scientific) supplemented with 10% fetal bovine serum (FBS; Hyclone Laboratories Inc., South Logan, UT, USA) and antibiotics at 37 °C and equilibrated with 95% humidified air with 5% CO_2_. 

### 2.3. Reagents

Antibodies against VEGFR2 (#2479), cyclin D1 (#2978S), cyclin B1 (#4135S), FAK (#3285, 1:1,000), phosphorylated (p)-FAK (Y397; #3283,), β1 integrin (#9699,), Akt (#9272,), p-Akt (S473; #9271, 1:1,000), caspase 3 (#9662S), cleaved PARP (#9541), p44/42 MAPK (ERK1/2; #9107), p-p44/42 MAPK (Thr 202/Tyr 204; #9101), vimentin (#3390), N-cadherin (#5296), β-catenin (#8480), GAPDH (#2118) and E-cadherin (#3195) were purchased from Cell Signaling Technology (Beverly, MA, USA). Antibodies against cyclin A (sc-271682) and FAK (sc-558) were purchased from Santa Cruz Biotechnology Inc. (Dallas, TX, USA). Cisplatin was purchased from Sigma (St. Louis, MO, USA).

### 2.4. Western Blotting

Protein extraction and immunoblotting were performed as previously described [10,13]. In brief, cells were lysed in protein lysis buffer (M-PERTM mammalian protein extraction buffer; Thermo Scientific, Rockford, IL, USA) and cellular debris was removed by centrifugation. Proteins were quantified, separated by sodium dodecyl sulfate polyacrylamide gel electrophoresis (SDS-PAGE), transferred to a nitrocellulose membrane, and immunoblotted using the indicated antibodies [10,13]. ImageJ (1.41o) was used for protein quantification.

### 2.5. RNA Interference and Plasmid Transfection

Short interfering RNA (siRNA) against human *FAK* (PTK2, M-003164-02-0005) and negative control siRNA against Firefly Luciferase (GL2) were purchased from Dharmacon Life Technologies (Cologne, Germany). Cells were transfected with 100 nM non-targeting or specific siRNA using Lipofectamine 2000 and Opti-MEM (both from Invitrogen, Carlsbad, CA, USA) according to the standard manufacturer’s protocol. 

Plasmid constitutive expressing a full-length wild-type FAK protein was obtained from Genecopoeia Inc. Cells were transfected with the appropriate amount of expression construct and control empty vector using Lipofectamine 2000 and Opti-MEM, according to Invitrogen’s recommendations [10]. Each experiment was repeated at least three times.

### 2.6. Cell Proliferation Assays

Cells (1 × 10^4^/well) were plated in each well of a 24-well plate. After three days of treatment with different doses of cisplatin, the cells were stained with 3-(4,5-Dimethylthiazol-2-yl)-2,5-diphenyltetrazolium bromide (MTT) solution (Sigma) and incubated for 1–2 h. The formazan crystals were then solubilized in dimethyl sulfoxide (DMSO) (Sigma) and absorbance was measured at 560 nm. Each experiment was repeated at least three times.

### 2.7. Cell Cycle Analysis

Cells were washed with phosphate buffered saline (PBS) and fixed with 70% alcohol at −20 °C for 24 h. Cells were collected by centrifugation, washed with PBS and stained with a DNA staining solution (50 μg/mL PI and 50 μg/mL RNaseA in PBS) for 30 min at room temperature. Cell cycle distribution was then evaluated using a flow cytometry (Accuri^TM^ C6, BD) and analyzed using the Accuri C6 software (BD).

### 2.8. Wound-Healing Assays

Different experimental groups of cells were seeded in 2-well silicone inserts (ibidi GmBH, Planegg, Germany). Cells were then incubated in culture medium at 37 °C in a 5% CO_2_ incubator for 24 h before removal of the inserts. Images were captured at 0 and 24 h. Each experiment was repeated at least three times.

### 2.9. Cell Invasion Assays

Cell invasion assays were performed as previously described [13]. In brief, cells were seeded in inserts placed in a Transwell in serum-free medium. Complete medium (500 μL DMEM containing 10% FBS) was added to the bottom chamber of the system. After 24 h of incubation, the cells were rinsed and stained with Giemsa (Sigma). Each experiment was repeated at least three times.

### 2.10. Immunofluorescence Staining

Cells were fixed with 4% paraformaldehyde, washed, and permeabilized with 0.2% Triton X-100 and 1% bovine serum albumin (BSA) in PBS. Cells were then incubated with primary antibodies overnight at 4 °C followed by incubation with secondary antibodies. Cells were washed three times with PBS and stained with 4′,6-diamidino-2-phenylindole (DAPI). Images were captured using a camera connected with a fluorescence microscope.

### 2.11. Specimens

Formalin-fixed, paraffin-embedded blocks of tissues from 69 patients with TNBC were obtained from the Department of Pathology, Kaohsiung Medical University Hospital, Kaohsiung, Taiwan. The Institutional Review Board approval for the use of these tissues in this study was given by the Research Ethics Committee of the Kaohsiung Medical University Hospital (IRB: KMUHIRB-E(I)-20170032) on 10 February 2017. The data were analyzed anonymously, and therefore no additional informed consent was required. All methods were performed in accordance with the approved guidelines and regulations of the Kaohsiung Medical University Hospital.

### 2.12. Immunohistochemistry (IHC) Staining

IHC staining was performed as previously described [35]. In brief, blocks of tissue samples embedded in paraffin were cut into 4-μm-thick sections, de-paraffinized and rehydrated. Antigen retrieval was achieved by autoclaving the sections at 121 °C for 10 min in a pH 6.0 antigen-retrieval solution (DAKO, Carpinteria, CA, USA). Endogenous peroxidase activity was blocked upon incubation in 3% hydrogen peroxide (Sigma) for 10 min. The sections were then incubated with the FAK primary antibody (Cell Signaling Technology) at room temperature for 1 h. The DAKO REAL™ EnVision™ Detection System EnVision (DAKO) was then applied for 1 h. Finally, the sections were incubated in 3′3-diaminobenzidine for 5 min, counterstained with Mayer’s hematoxylin and mounted. Negative controls were prepared by replacing the primary antibodies with non-immune serum.

### 2.13. Scoring

FAK expression in samples from different patients was scored according to Yin et al. [13]: samples were scored based on the intensity of signal (0, 1+, 2+ and 3+), and the proportion of positive cells (0 ≤ 10%, 1 = 10–25%, 2 = 25–50%, 3 ≥ 50%). The staining index was calculated as the product of the intensity of signal and the proportion of positive cells, and scores ranged from 0 to 9. A score ≤ 4 indicated low expression of FAK and a score ≥ 6 indicated high expression.

### 2.14. Statistical Analysis

The expression of FAK in TNBC tissues as determined by IHC staining was compared using the Chi-square test. To evaluate the use of FAK for TNBC prognosis, survival curves were obtained using the Kaplan-Meier method. A Cox proportional hazards model was used to evaluate univariate comparisons of overall survival with the clinicopathologic variables. A two-tailed Student’s t test was used to compare differences between groups. A *p* value < 0.05 was considered to indicate statistically significant differences between groups. All statistical analyses were performed using the SPSS 19.0 software (IBM Corp., Armonk, NY, USA).

## 3. Results

### 3.1. FSS Alters the Morphology, Cell Cycle, Proliferation and Survival of TNBC Cells

**Figure 1:**
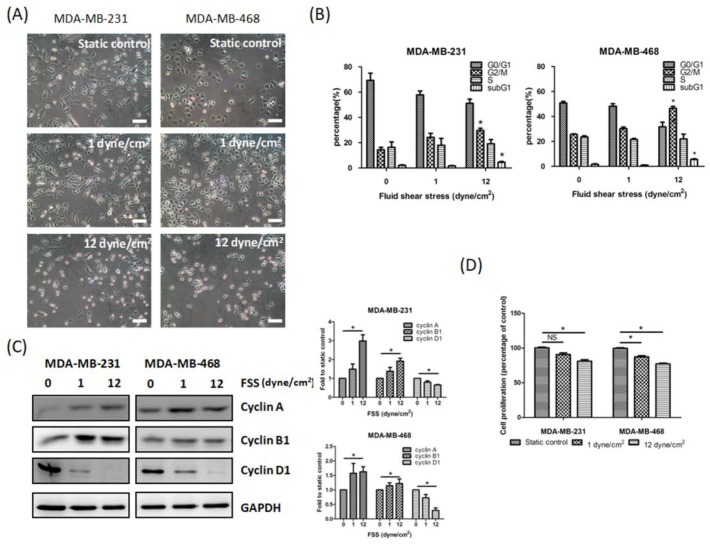
Fluid shear stress (FSS) affects the morphology, cell cycle, proliferation and survival of MDA-MB-231 and MDA-MB-468 triple-negative breast cancer (TNBC) cells. (**A**) Effect of low and high FSS for 24 h on the morphology of MDA-MB-231 and MFA-MB-468 TNBC cells. Scale bar: 100 μm. (**B**) Cell cycle distribution in relation to FSS. (**C**) Representative western blot revealed that FSS affects the levels of several cell cycle-related proteins in MDA-MB-231 and MFA-MB-468 TNBC cells. (**D**) Low and high FSS affect cell proliferation (indicated as percentage of static control) in both TNBC cell lines. Data from at least three experiments were used for statistical analysis and * *p* < 0.05.

Tumor cells undergo a different shear stress when they are adherent on the tissue or move to a distant organ, because of the interstitial or blood flow, respectively. Therefore, we used a PPFC to generate the physiological range of FSS (1 and 12 dyn/cm^2^) on TNBC (MDA-MB-231 and MDA-MB-468) cells. At first, we characterized the effect of FSS on TNBC cell morphology. Following low FSS (1 dyn/cm^2^) for 24 h, both TNBC cell lines showed a more pronounced elongated morphology and increased spread area compared to the static control. Contrarily, after 24 h of high FSS (12 dyn/cm^2^) the cells showed a rounder shape than the static control (Figure 1A). Therefore, FSS altered the morphology of TNBC cells.

Previous studies indicated that FSS regulates cell cycle and proliferation in several cancer cells [7,8,10]. Here, we investigated whether FSS could also affect these processes in TNBC cells. In accordance with our previous study [10], high FSS for 24 h significantly increased the percentage of G_2_/M cells from 15% to 25% and from 25% to 45% in MDA-MB-231 and MFA-MB-468 cells, respectively. We found that low FSS also increased the percentage of G_2_/M cells but the results were not significant. We also noted that high FSS increased levels of the regulatory proteins of cell cycle cyclin A and B1, but decreased cyclin D1 levels (Figure 1C). In addition, high FSS, but not low FSS, induced significant cytotoxicity (subG1 phase) in both TNBC cell lines (Figure 1D). We also found that low and especially high FSS inhibited cell proliferation (10% and 25% decrease 72 h after low and high FSS treatment, respectively) in both MDA-MB-231 and MDA-MB-468 cells (Figure 1D).

### 3.2. FSS Regulates Cell Motility by Altering The Expression of EMT-Related Proteins and FAK in TNBC Cells

**Figure 2:**
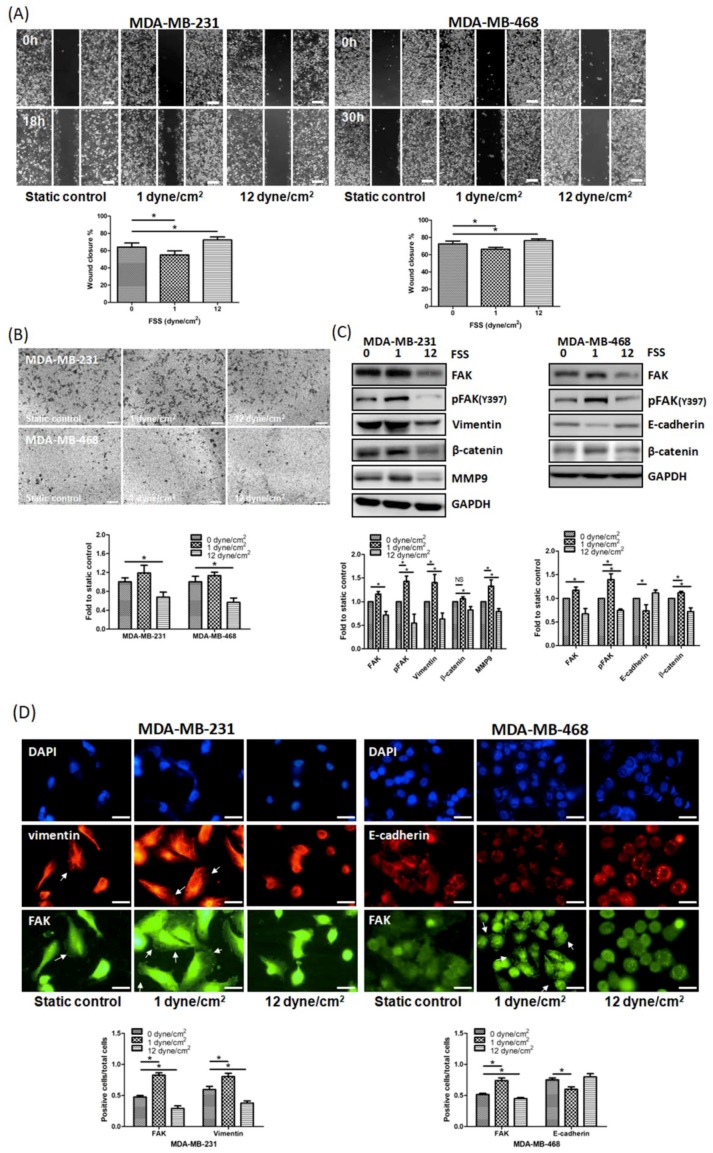
FSS affects cell motility by altering the expression of epithelial-mesenchymal transition (EMT)-related proteins and focal adhesion kinase (FAK) in TNBC cells. (**A**) Wound healing assays showed that high FSS inhibited migration, while low FSS promoted it in MDA-MB-231 and MDA-MB-468 cells. Scale bar: 100 μm. (**B**) Cell invasion assays showed that high FSS reduced the number of invasive cells, while low FSS promoted it in MDA-MB-231 and MDA-MB-468 cells. Scale bar: 200 μm. (**C**) FSS altered the expression of several EMT-related proteins and FAK in MDA-MB-231 and MDA-MB-468 cells. (**D**) Representative immunofluorescence images of FAK (green)/vimentin (red) or FAK (green)/E-cadherin (red) after low/high FSS treatment in MDA-MB-231 and MDA-MB-468 cells. Vimentin structure and focal adhesions are indicated with white arrow heads. Scale bar: 10 μm. Data from at least three experiments were used for statistical analysis and * *p* < 0.05.

Our previous study suggested that FSS regulates the cytoskeleton through the FAK signaling pathway [10]. Additionally, previous studies indicated that FAK upregulates the EMT in several cancer cell lines [23,24]. Therefore, we decided to investigate whether, in TNBC cells, FSS regulates cell motility through FAK and EMT-related proteins. High FSS significantly inhibited cell migration and invasion, while low FSS had opposite effects (Figure 2A,B). Western blotting and immunofluorescence assays showed that high FSS decreased FAK and p-FAK expression, while low FSS increased them (Figure 2C,D). In addition, in both TNBC cell lines, high FSS inhibited the expression of several EMT-associated proteins such as β-catenin and vimentin, while low FSS induced their expression (Figure 2C,D). In MDA-MB-468 cells, we also observed that the expression of E-cadherin was induced by high FSS but inhibited by low FSS (Figure 2C,D). These data strongly indicate that the migration and invasion abilities affected by FSS are related to the expression of FAK and EMT-related proteins.

### 3.3. Downregulation of FAK by FSS Correlates with Enhanced Cisplatin Sensitivity

**Figure 3:**
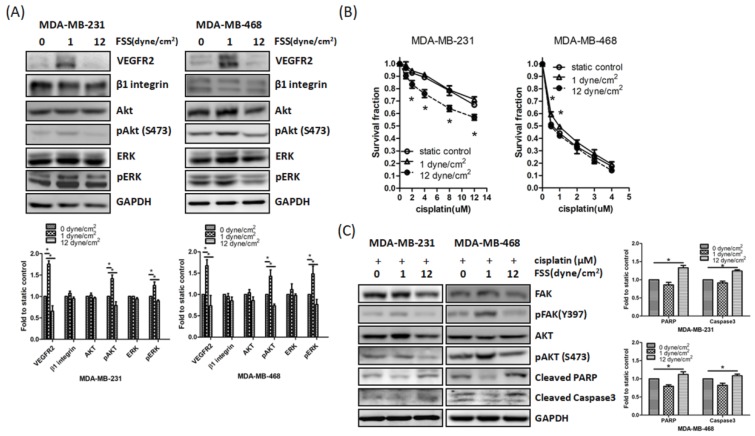
FSS affects the cisplatin sensitivity via FAK in MDA-MB-231 and MDA-MB-468 cells. (**A**) Low/high FSS affected several protein expressions in MDA-MB-231 and MDA-MB-468 cells. (**B**) Low/high FSS affected the cytotoxicity of cisplatin in MDA-MB-231 and MDA-MB-468 cells. (**C**) Representative western blot showed low/high FSS affected cisplatin-induced apoptosis in MDA-MB-231 and MDA-MB-468 cells. Data from at least three experiments were used for statistical analysis and * *p* < 0.05.

The data reported above indicated that different degrees of FSS affected FAK expression in TNBC cells. Prior studies have suggested that FAK is associated with drug resistance [36] and our previous study indicated that high FSS upregulates radiosensitivity via the FAK pathway in colon cancer cells [10]. Therefore, we hypothesized that FSS affects the fate of TNBC cells via the FAK signaling pathway. We found that the expression of VEGFR2, p-Akt, and p-ERK was inhibited by high FSS (Figure 3A). In addition, exposure to high FSS for 24 h prior to cisplatin treatment significantly increased the cytotoxicity of cisplatin in MDA-MB-231 cells (Figure 3B). On the contrary, low FSS increased the expression of the aforementioned proteins and reduced the cytotoxicity of cisplatin treatment in MDA-MB-468 cell (Figure 3A,B). Western blot assays showed that the levels of the cleaved forms of PARP and caspase 3 increased when TNBC cells were treated with high FSS and cisplatin and decreased when the cells were treated with low FSS (comparing each condition to static controls; Figure 3C). These observations suggested that FAK might be an important mediator of the cisplatin-sensitizing effect of FSS.

### 3.4. FSS Modulated Cell Motility and Cisplatin-Induced Cell Death *via* FAK-VEGFR2 Axis Pathway

**Figure 4:**
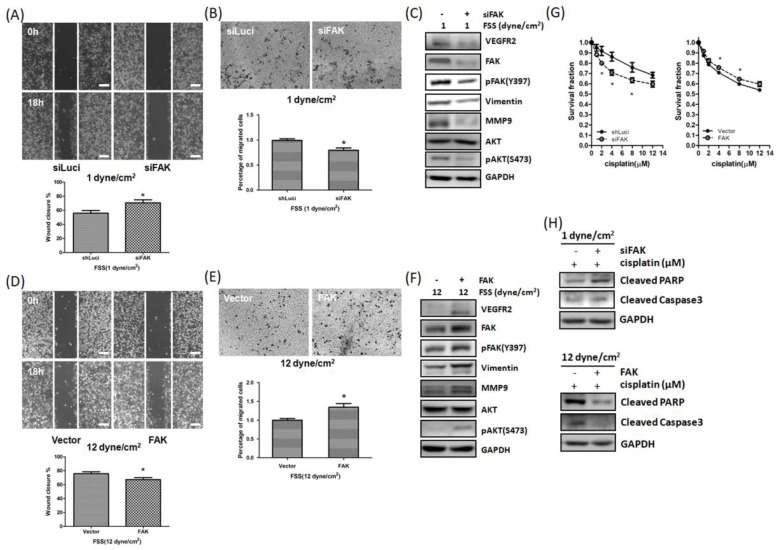
FAK plays a role in FSS-mediated cell motility and cisplatin resistance. (**A**,**B**) Knockdown of *FAK* counteracted the induction of cell migration (A) and invasion (B) promoted by low FSS in MDA-MB-231 cells. (**C**) Low FSS promoted VEGFR2, FAK, p-FAK, vimentin, MMP-9 and p-AKT expression, while knockdown of FAK counteracted this effect. (**D**,**E**) Overexpression of FAK counteracted the inhibition of migration (D) and invasion (E) promoted by high FSS in MDA-MB-231 cells. (**F**) High FSS decreased VEGFR2, FAK, p-FAK, vimentin, MMP-9 and p-AKT expression, while overexpression of FAK counteracted this effect. (**G**) Knockdown of *FAK* increased the cytotoxic effect of cisplatin on MDA-MB-231 cells induced by low FSS. In contrast, overexpression of FAK decreased the sensitivity to cisplatin induced by high FSS. (**H**) Representative western blot showing that knockdown of *FAK* increased cisplatin-induced apoptosis, which was inhibited by low FSS in MDA-MB-231 cells; overexpression of FAK decreased cisplatin-induced apoptosis promoted by high FSS. Data from at least three experiments were used for statistical analysis and * *p* < 0.05. Scale bar in (A,D): 100 μm. Scale bar in (B,E): 200 μm.

To further clarify the role of FAK in FSS-regulated cell motility and cisplatin sensitivity in TNBC cells, we next knocked down or overexpressed FAK, exposed the cells to low or high FSS, and assessed the cells’ migration, invasion, and sensitivity to cisplatin. The increased migration and invasion, induced by low FSS, were inhibited by knockdown of *FAK* in MDA-MB-231 cells (Figure 4A,B). In addition, western blot assays indicated that the effect of low FSS on the expression of VEGFR2, p-Akt and EMT-related proteins could be reversed by *FAK* knockdown (Figure 4C). We also found that knockdown of *FAK* could overcome the resistance to cisplatin induced by low FSS (Figure 4G). Conversely, overexpression of FAK efficiently rescued the inhibition of migration and invasion and increased the cytotoxic effect of cisplatin induced by high FSS (Figure 4D–G). Additionally, western blot showed that knockdown of *FAK* reversed the low FSS-induced resistant effect on cisplatin (Figure 4H, top). Conversely, overexpression of *FAK* in MDA-MB-231 cells decreased the high FSS-induced sensitizing apoptosis effect on cisplatin (Figure 4H, bottom). Our IHC staining from patients with TNBC also showed that the expression of VEGFR2 was parallel with FAK (Supplementary Materials, Figure S1). These results indicated that FAK plays an important role in FSS by regulating several malignant characteristics in TNBC cells.

### 3.5. FSS Affects CTCs via the FAK Signaling Pathway

**Figure 5:**
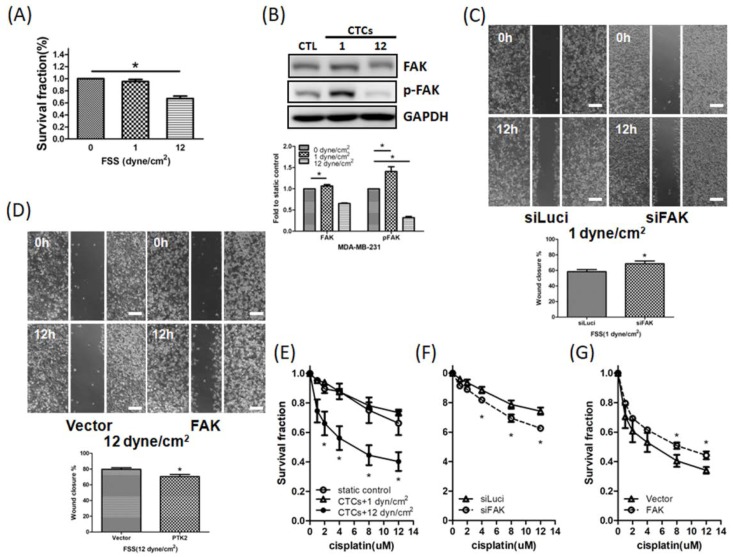
FSS affects the proliferation, survival, migration and drug sensitivity in MDA-MB-231 CTCs via FAK. (**A**) High FSS decreased the proliferation in MDA-MB-231 CTCs. (**B**) Low FSS increased FAK and p-FAK expression, while high FSS decreased them. (**C**) Knockdown of *FAK* inhibited CTCs’ migration promoted by low FSS. Scale bar: 100 μm. (**D**) FAK overexpression increased CTCs’ migration decreased by high FSS. Scale bar: 100 μm. (**E**) High FSS, but not low FSS, increased the cytotoxicity of cisplatin in CTCs. (**F**) Knockdown of *FAK* increased the cytotoxicity of cisplatin in CTCs induced by low FSS. (**G**) Overexpression of FAK decreased the sensitivity of cisplatin induced by high FSS. Data from at least three experiments were used for statistical analysis and * *p* < 0.05.

Next, we investigated whether FSS affects CTCs by using our modified circulating system. As shown in Figure 5A, high FSS negatively affected the number of CTCs, while low FSS did not have this effect. Similar to what was described above for adherent cells, western blotting showed that high FSS decreased FAK and p-FAK expression, while low FSS increased it (Figure 5B). Low FSS, but not high FSS, promoted migration of CTCs (Figure 5C,D). In addition, high FSS sensitized CTCs to cisplatin (Figure 5E), while low FSS did not have this effect. Knockdown of *FAK* in CTCs exposed to low FSS inhibited migration and increased the cytotoxicity of cisplatin (Figure 5C right panel and Figure 5F). In contrast, overexpression of FAK in CTCs exposed to high FSS promoted migration and decreased the cytotoxicity of cisplatin (Figure 5D right panel and Figure 5G). Therefore, FSS regulated the motility and survival of CTCs via FAK signaling.

### 3.6. Relationship between FAK Levels and Clinicopathological Parameters

**Figure 6:**
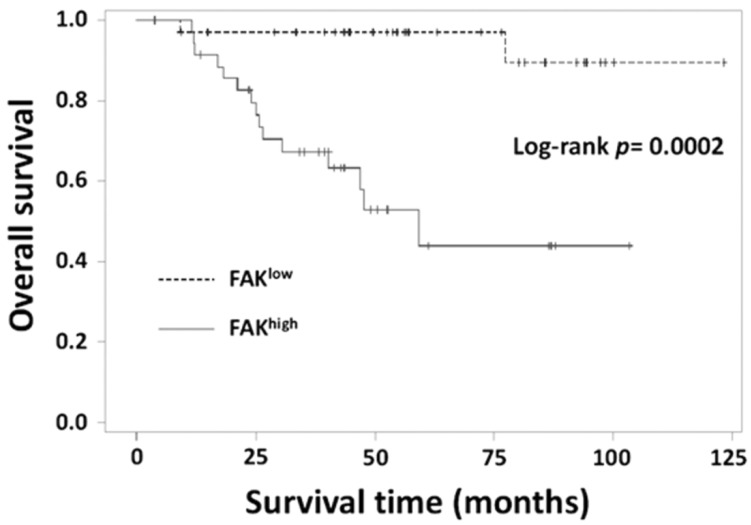
Kaplan-Meier survival curves for patients with TNBC. Patients’ survival was significantly associated with FAK expression.

High expression of FAK has been reported in many cancer types and is related to metastasis formation. Therefore, we investigated whether the same happened in TNBC. We analyzed 69 patients with TNBC with an average age of 51.9 ± 11.2 years (range 27–81 years). We first investigated whether FAK expression correlated with the patients’ clinicopathological parameters and survival. Table 1 indicates that FAK expression was significantly associated with the nodal stage and death (*p* = 0.015 and 0.0006, respectively). We then performed Kaplan-Meier analysis to compare the survival of patients with high and low expression of FAK. The average disease-specific survival time (from time of diagnosis to time of death due to TNBC) in patients with high FAK levels was 51.7 ± 28.6 months, a value significantly lower than that in patients with low FAK expression (*p* < 0.0001, Figure 6). Univariate analysis (Table 2) revealed that the overall survival of the patients was significantly associated with FAK expression (*p* = 0.0188), nodal stage (*p* = 0.0107), and grade (*p* = 0.0263). Multivariate Cox regression analysis of FAK expression, age, tumor grade and TNM stage, showed that FAK expression was a significant independent predictor of overall survival (*p* = 0.0445). Taken together, these data suggest that FAK is a biomarker for TNBC survival and nodal stage.

## 4. Discussion

Cancer cell invasion and metastasis are regarded as the main causes of cancer-related mortality. Many studies have suggested that metastasis, drug resistance, and recurrence in cancer are regulated by the tumor microenvironment [37]. Although the role of many biochemical factors in tumorigenesis has been widely investigated, the impact of physical factors in the same process is still unclear. In recent years, the effect of some physical factors (such as FSS, hardness and stiffness) on normal/tumor cells has become evident and is reshaping our knowledge of the mechano-transduction mechanisms [18,37,38]. However, the impact of flow-associated physical forces, such as FSS, on tumor cell behavior remains unclear.

Many studies have demonstrated that FSS regulates several functions in normal and tumor cells [3,7,8,9,10,11,32,33,39,40,41,42,43]. However, the influence of different degrees of FSS on tumor cells is not clear. TNBC, a subtype of breast cancer, is more aggressive than other breast cancer subtypes. The incidence of early lymph node involvement and distant metastasis is higher in TNBCs than in other breast cancer subtypes [13,44], suggesting that TNBC cells might overcome the hemodynamic stimulation better than non-TNBC cells. Thus, we chose TNBC cells as an in vitro model to study the effects of FSS. Our previous study indicated that FAK regulates the radiosensitivity induced by FSS [10]; thus, we hypothesized that FSS regulates processes such as cell cycle, cell proliferation, migration, invasion, and drug sensitivity in TNBC through FAK signaling.

Our results demonstrated that FSS affects cell morphology and proliferation and increases the percentage of G_2_/M cells (Figure 1A–D). These results are similar to those of other investigators [7,8,10]. Although the increasing of G_2_/M phases was induced by both low and high FSS, there was no significant difference in low FSS increasing G_2_/M accumulation. These results seemed to be bypassed by FAK upregulation [45]. Furthermore, our functional assays and western blot demonstrated that FSS regulates cell migration and invasion through EMT-related proteins and the FAK signaling pathway (Figure 2). The proliferation rates of MDA-MB-231 and MDA-MB-468 cells were decreased by both low and high FSS, however, the doubling time of both cells was more than 30 h. Therefore, the migration and invasion induced by low/high FSS are not affected by cell proliferation.

In our study, while high FSS inhibits both total and phospho-FAK expression, low FSS tends to only promote FAK phosphorylation without significant changes in total FAK protein level. In our previous study, we indicated that downregulation of FAK by high FSS is via ubiquitination mediated proteosomal degradation, and other studies have also indicated that FSS promotes several protein degradation in endothelial or cancer cells [10,46,47] Therefore, we supposed that the inhibition of total and phosphor-FAK by high FSS in TNBC cells could also be related to protein stability and degradation. Conversely, low FSS significantly increased the phosphor-FAK expressions in MDA-MB-231 and MDA-MB-468 cells. According to several prior studies, FSS could promote cell motilities and mortalities through the integrins/FAK-related signaling pathway [3,10,13,33,41]. Therefore, phosphorylation of FAK could be activated by integrins and is needed to drive the downstream signaling transduction. In addition, prior studies have indicated that E-cadherin was regulated via the FAK signaling pathway in several cancer cells [48,49]. Therefore, we supposed that the regulation of E-cadherin in high/low FSS could also occur via FAK in our study. Whether other factors are involved in this process needs to be clarified.

Prior studies have demonstrated that FAK regulates drug sensitivity in many cancer cells [26,27,50,51,52] and FSS could regulate several cell functions via the FAK pathway [3,10,32,33]. Thus, we hypothesized that changes in FAK expression induced by FSS regulate the cisplatin sensitivity of TNBC cells. Our results indicated that high FSS increased the cytotoxicity of cisplatin in MDA-MB-231 cells and low FSS reduced the cytotoxicity of cisplatin in MDA-MB-468 cells (Figure 3). However, high and low FSS did not have the same effects in MDA-MB-468 and MDA-MB-231 cells, respectively. A previous study has indicated that MDA-MB-231 cells are more resistant to cisplatin than MDA-MB-468 cells [53]. FAK expression in MDA-MB-231 is also higher than MDA-MB-468 cells. We think that levels of FAK might play a role in determining cell response to cisplatin. Therefore, even though low FSS upregulated FAK expression, the increased FAK expression did not increase the resistance to cisplatin of MDA-MB-231 cells; conversely, even though high FSS downregulated FAK expression, the reduction of FAK levels was not sufficient to increase the sensitivity to cisplatin of MDA-MB-468 cells. Taken together, FAK downregulation or overexpression, combined with low or high FSS, respectively, proved the role of FAK in the regulation of FSS-mediated cell migration, invasion, and drug sensitivity (Figure 4). Therefore, FAK seems to be a fluid mechanosensor that regulates the cell motility and survival induced by different degrees of FSS.

Previous studies have indicated that VEGFR2 upregulates several downstream EMT-related genes and MMPs, which promote cell motility in cancer cells [54,55,56,57]. On the other hand, cytokines and growth factors also participate in VEGFR2-regulated cell proliferation/survival in cancer cells. Therefore, targeting VEGFR2 in endothelial/malignant cells could be an effective treatment in cancer [58]. Here, our in vitro results indicate that FSS regulates VEGFR2 expression via FAK (Figure 2). Knockdown of *FAK* counteracted the increase in VEGFR2 and downstream MMP9, pAKT and pERK induced by low FSS (Figure 4). Additionally, IHC staining of samples from patients with TNBC showed that the expression of VEGFR2 parallels that of FAK (Supplemental Figure S1). These data are similar to those of Sun et al., indicating that FAK and its kinase activity regulate *VEGFR2* transcription [59]. The present study also suggests that FAK inhibitors could reduce metastasis formation and angiogenesis, in agreement with the findings of Lv and colleagues [50]. Thus, the inhibition of FAK could be a strategy which is relevant in the design of anti-cancer drugs and anti-angiogenic therapies.

A previous study suggested that the use of a PPFC to study how FSS affects cellular functions in adherent cells is not ideal, because tumor cells are circulating cells, which experience FSS in the blood flow [11]. Therefore, we also studied the influence of FSS on CTCs using our circulatory system. Our results indicated that low FSS also induces slight resistance to cisplatin and promotes cell migration in TNBC CTCs. On the other hand, high FSS reduces CTCs’ proliferation and induces cell death, sensitizes the cells to cisplatin and inhibits cell migration. Downregulation of *FAK* in cells exposed to low FSS increases the cytotoxicity of cisplatin and inhibits cell migration, suggesting that FAK plays an important role in the regulation of the fate and the migration of CTCs. Tumor cells leaving a primary tumor are subjected to FSS in the blood and lymphatic vessels. However, tumor cell metastases occur more frequently in some organs (such as lung and liver) than in others (such as heart or large arterial walls), suggesting that the success of metastasis is possibly related to the different degrees of FSS. Thus, the modulation of FSS should be considered for the development of future cancer treatments.

Our clinical data showed that high FAK expression significantly correlated with the nodal stage and low survival rate (Table 1) and the average disease-free survival in patients with high FAK expression was significantly lower than that in patients with low FAK expression (Figure 6). Univariate and multivariate Cox regression analyses showed that FAK expression was significantly associated with survival and nodal stage, and was a significant predictor of overall survival (Table 2). Therefore, our data suggest that FAK is a potential prognostic marker of survival in patients with TNBC and is an indicator of TNBC cells’ metastasis.

## 5. Conclusions

In summary, we used high and low FSS to investigate whether FSS could regulate cellular behavior and drug sensitivity in TNBC cells and further investigated the involvement of FAK signaling in these processes. Our data demonstrate the importance of FAK in FSS-mediated cell migration and drug sensitivity in TNBC cells. Additionally, we showed that FAK levels correlate with the nodal stage and that FAK is a prognostic marker in patients with TNBC.

## Figures and Tables

**Table 1:** Relationship between FAK expression and the clinicopathological characteristics of patients with TNBC (*n* = 69).

Parameters	*n*	FAK, *n* (%)		*p*-Value
		Low	High	
Total	69	33 (47.83)	36 (52.17)	
Age, *n* (%)				0.8769
≤40 years	8	4 (14.29)	4 (12.90)	
>40 years	51	24 (85.71)	27 (87.10)	
Size, *n* (%)				0.3458
≤2.0 cm	23	13 (39.39)	10 (28.57)	
>2.0 cm	45	20 (60.61)	25 (71.43)	
Grade, *n* (%)				0.8793
I/II	29	12 (48.00)	17 (50.00)	
III	30	13 (52.00)	17 (50.00)	
Tumor stage, *n* (%)				0.4298
T1	28	15 (45.45)	13 (36.11)	
T2/T3	41	18 (54.55)	23 (63.89)	
Nodal stage, *n* (%)				0.015 *
N0	42	25 (75.76)	17 (47.22)	
N1/N2/N3	27	8 (24.24)	19 (52.78)	
Metastatic stage, *n* (%)				0.8817
M0	59	28 (84.85)	31 (86.11)	
M1	10	5 (15.15)	5 (13.89)	
Tumor recurrent, *n* (%)				0.3348
Absent	68	33 (100.00)	35 (97.22)	
Present	1	0 (0.00)	1 (2.78)	
Survival status, *n* (%)				<0.01 *
Survival	52	31 (93.94)	21 (58.33)	
Death	17	2 (6.06)	15 (41.67)	

Statistical analysis was performed using the Chi-squared test. * Statistically significant (*p* < 0.05).

**Table 2:** Univariate and multivariate analyses of clinicopathological independent prognostic factors for survival of patients with TNBC (*n* = 69).

Factors	Univariate		Multivariate	
	HR (95%CI)	*p*-Value	HR (95%CI)	*p*-Value
FAK expression		0.0188 *		0.0445 *
Low	1.0		1.0	
High	6.174 (1.352–28.195)		5.393 (1.042–27.917)	
Age, *n* (%)		0.4307		0.1774
≤40 years	1.0		1.0	
>40 years	2.277 (0.294–17.646)		4.508 (0.505–40.200)	
Size, *n* (%)		0.0846		
≤2.0 cm	1.0			
>2.0 cm	6.038 (0.783–46.581)			
Grade		0.0263 *		0.0156 *
I/II	1.0		1.0	
III	4.386 (1.191–16.158)		6.470 (1.425–29.388)	
Tumor stage, *n* (%)		0.0494 *		0.7186
T1	1.0		1.0	
T2/T3	4.533 (1.004–20.464)		1.397 (0.227–8.620)	
Nodal stage, *n* (%)		0.0107 *		0.0443 *
N0	1.0		1.0	
N1/N2/N3	5.368 (1.476–19.515)		4.688 (1.040–21.128)	
Metastatic stage, *n* (%)		0.5432		0.3667
M0	1.0		1.0	
M1	1.495 (0.409–5.460)		0.506 (0.115–2.221)	

Statistical analysis was performed using Cox multivariate analysis. * Statistically significant (*p* < 0.05).

## Supplementary Materials

The following are available online at http://www.mdpi.com/2077-0383/8/1/38/s1, Figure S1: Expression of FAK and VEGFR2 in TNBC tissues.
