# Supplementary material for: FAK is Required for Tumor Metastasis-Related Fluid Microenvironment in Triple-Negative Breast Cancer
Source: J Clin Med. 2019 Jan 2;8(1):38. doi: 10.3390/jcm8010038 (PMC6352244; doi:10.3390/jcm8010038)
Supplement: Supplementary file 1 [file jcm-08-00038-s001.pdf]

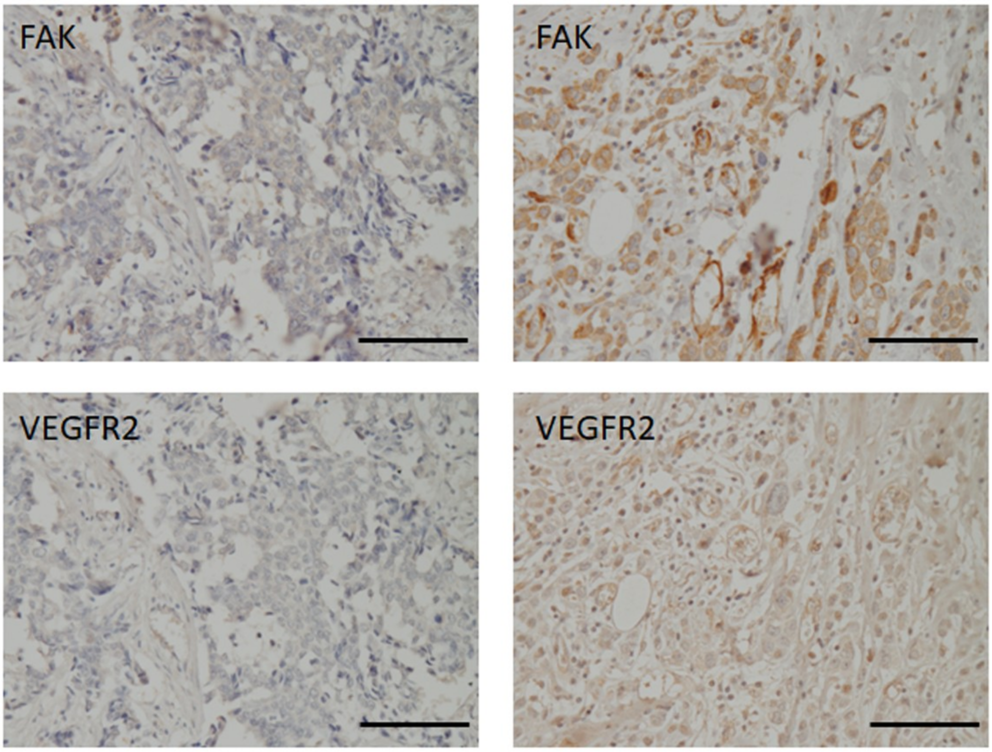

Figure S1. Expression of FAK and VEGFR2 in TNBC tissues. Tissue samples were classified as negative (left) or positive (right) for FAK and VEGFR2 expression according to the intensity of IHC staining observed in the cytoplasm (original magnification: ×200)
